# The effectiveness of preliminary traction in the treatment of congenital dislocation of the hip

**DOI:** 10.1186/s10195-021-00586-8

**Published:** 2021-06-27

**Authors:** Pasquale Farsetti, Kristian Efremov, Alessandro Caterini, Martina Marsiolo, Fernando De Maio, Ernesto Ippolito

**Affiliations:** Department of Clinical Science and Translational Medicine, Section of Orthopaedics and Traumatology, University of Rome “Tor Vergata”, Rome, Italy

**Keywords:** Developmental dislocation of the hip (DDH), Congenital dislocation of the hip (CDH), Preliminary traction, Avascular necrosis (AVN)

## Abstract

**Background:**

Historical papers on the treatment of congenital dislocation of the hip suggest the use of preliminary traction to facilitate closed reduction or to decrease the risk of avascular necrosis (AVN) of the femoral head. In the 1980s, some authors questioned the role of preliminary traction and suspended its use, yielding satisfactory results. Since then, several studies called into question this method, and some authors have continued to recommend preliminary traction while other authors have discouraged its use.

**Materials and methods:**

We reanalysed the full set of radiographs of 71 hips (52 patients) surgically treated by a medial approach after 4 weeks of preoperative longitudinal traction. The mean age at operation was 16 months. Before and after traction, the height of the dislocation was graded according to the Gage and Winter method. The hips were divided into two groups: group 1, in which the traction was effective, and group 2, in which the traction was not effective. These two groups were statistically analysed regarding the severity of the dislocation, the age of the patient at surgery and the incidence of AVN.

**Results:**

Preliminary traction was effective in 48 hips (68%, group 1), while it was not effective in the remaining 23 (32%, group 2). The effectiveness of preliminary traction was statistically related to the height of the dislocation and to the age of the patient at surgery, with traction being less effective in more severe dislocations and in older children. The incidence of AVN was statistically lower in group 1 than in group 2.

**Conclusions:**

In our study population, despite not having a control group, preliminary traction—when effective—seemed to reduce the incidence of AVN in patients surgically treated for congenital dislocation of the hip. The effectiveness of the traction was influenced by the severity of the dislocation and the age of the patient; it worked better for less severe dislocations and in younger children. To reduce hospital costs, traction should be applied at home.

**Level of evidence:**

3.

## Introduction

Developmental dysplasia of the hip (DDH) is an important topic in paediatric orthopaedics [1, 2]. In DDH, the femoral head and acetabulum are misaligned, and the goal of treatment is to obtain a stable concentrically reduced hip joint as soon as possible. Early diagnosis and treatment are crucial to obtaining better results and avoiding surgical procedures [3–5]. An abduction brace is the standard treatment for children younger than 6 months, with a success rate of greater than 90% [6, 7]. However, in some cases, conservative treatment fails or the diagnosis is delayed and a hip subluxation or dislocation occurs. In those cases, closed reduction under general anaesthesia or open reduction is indicated [3, 8–10]. The use of preliminary traction to facilitate closed reduction or to decrease the risk of avascular necrosis (AVN) is still debated [11–13]. In the 1980s, some authors questioned the role of preliminary traction in the treatment of congenital dislocation of the hip (CDH) and suspended its use, reporting satisfactory results in patients treated for CDH without preliminary traction, with a low incidence of AVN [14, 15]. Since then, there has been no unanimous consensus on the use of preliminary traction [16–23]. The aim of the present study was to determine the real efficacy of traction in pulling down the dislocated hip, and to report the effectiveness of preliminary traction at decreasing the incidence of AVN in a series of children 3–36 months old who were surgically treated for CDH by the medial approach.

## Materials and methods

In 2015 we published an article reporting the long-term follow-up for a series of 71 hips (52 patients) surgically treated by a medial approach after 4 weeks of preoperative traction [24]. We reanalysed the full set of radiographs for every patient from diagnosis to follow-up. All patients were put in traction before surgical reduction for 4 weeks. Bilateral cases were treated in two consecutive stages because our traction system does not allow simultaneous traction on both limbs. Before and after traction, the height of the dislocation was graded according to the Gage and Winter method [25]. The authors described four stations to identify the height of dislocation of the proximal femoral epiphysis (the minus-one, zero, plus-one and plus-two stations). The medial corner of the metaphysis and the nucleus of the femoral head, when it was present, represented the reference points. At the minus-one station, the medial corner of the metaphysis was superior to the Hilgenreiner line; at the zero station, the medial corner of the metaphysis was at the level of the triradiate cartilage; at the plus-one station, the medial corner of the metaphysis was below the Hilgenreiner line as well as the centre of the capital epiphysis (normal hip); at the plus-two station, the capital epiphysis was below the normal hip.

**Fig. 1:**
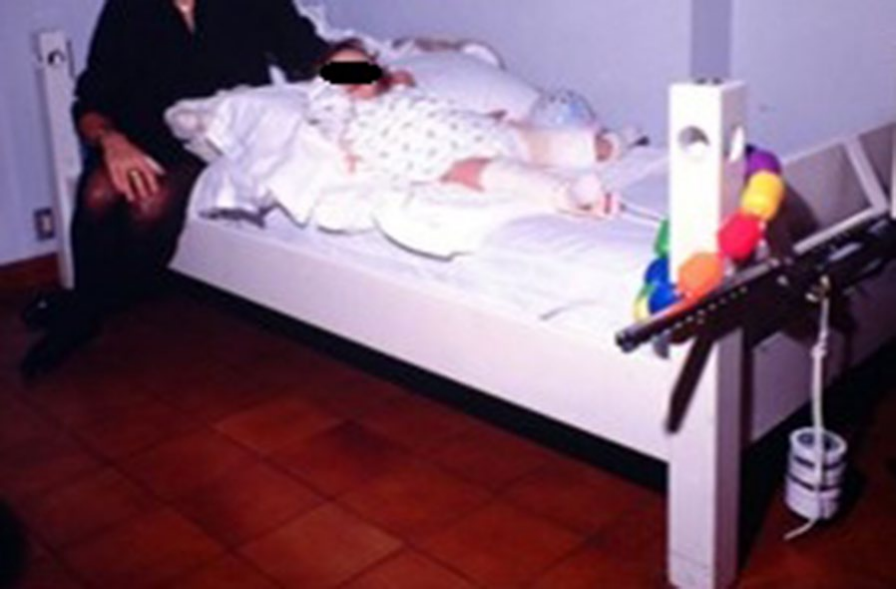
Preliminary traction system applied at home in an 18-month-old patient affected by right congenital dislocation of the hip. The child was anchored to the bed through a hip spica cast applied on the contralateral side

**Fig. 2:**
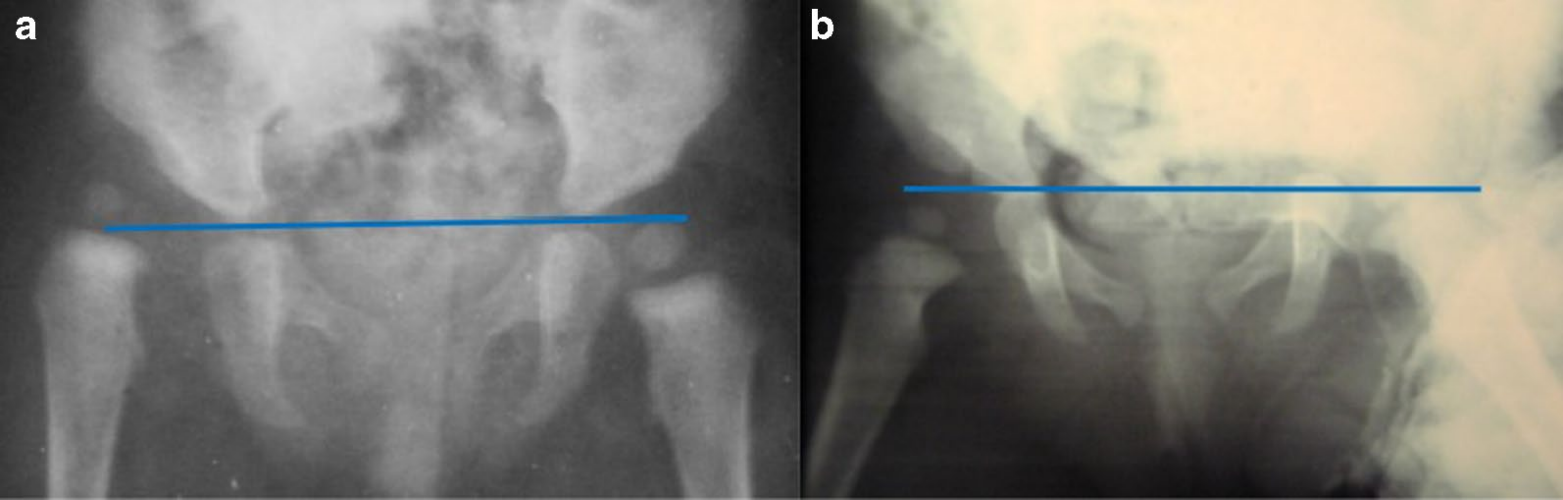
Radiographic examination of congenital dislocation of the right hip in a 14-month-old child before starting treatment by preliminary traction. The affected hip was classified as being at the minus-one station according to the Gage and Winter method (**a**). After 4 weeks, X-ray showed the effectiveness of the traction, as it had pulled the femoral head down to the plus-one station (group 1) (**b**)

**Fig. 3:**
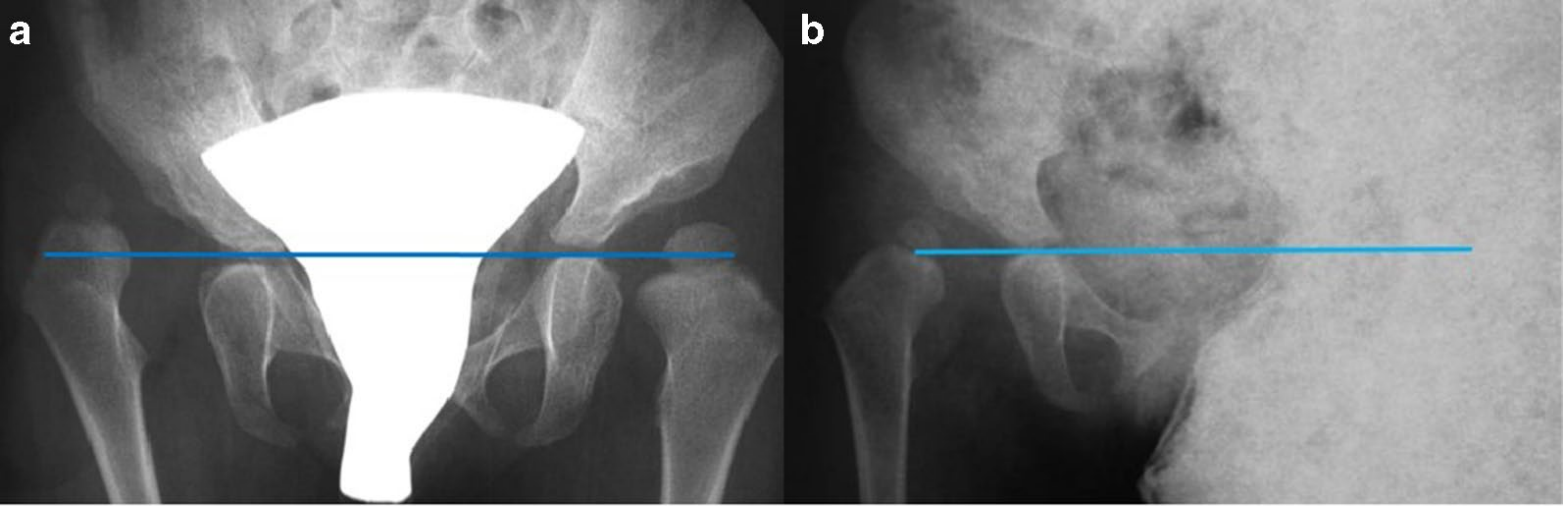
Radiographic examination of congenital dislocation of the right hip in a 30-month-old child before starting treatment by preliminary traction. The affected hip was classified as being at the minus-one station according to the Gage and Winter method (**a**). After 4 weeks, X-ray showed that the femoral head was at the zero station, so the traction was considered to be ineffective (group 2) (**b**)

Regarding the application of traction, we adopted the method described by Lehman et al. [26], who proposed a longitudinal traction system, in order to obtain effective traction and better verify its efficacy through radiographic monitoring. We applied a hip spica cast to the contralateral side to anchor the child to the bed, avoiding progressive sliding of the patient downwards. The child was anchored to the bed by means of two strings passing through two rings incorporated into the cast. Skin traction of one-sixth of the patient’s weight was applied to the dislocated hip, and the weight was gradually increased to a maximum of two-thirds of the body weight, according to the staged radiographic monitoring (Fig. 1). The direction of traction was about 20° of flexion and 20° of abduction. All the children were checked every day, both in the hospital and at home, by an orthopaedist on our staff or by a senior resident, to clinically monitor the patients, the traction system. Moreover, in the hospital, the nurses were in charge of taking care of the children; at home, the traction system was monitored by the parents, who were in telephone contact with our residents in case of any problems. The effectiveness of the traction was verified by once-weekly radiographic examination of the pelvis in bed. Traction was considered to be effective when the femoral metaphysis was pulled down to the plus-one or plus-two station. Traction was always stopped at the end of the 4th week. To reduce hospital costs, traction was applied and maintained at home in about half of our patients. The real effectiveness of traction at pulling down the dislocated hip was radiographically evaluated according to the Gage and Winter method, and the hips were divided into two groups: group 1, in which the traction was effective (Fig. 2), and group 2, in which the traction was not effective (Fig. 3). These two groups were statistically analysed regarding the initial severity of the dislocation, the age of the patient at surgery, and the incidence of AVN of the proximal femoral epiphysis according to the classification of Bucholz and Ogden [27] as modified by Morcuende et al. [4].

All methods were carried out in accordance with relevant guidelines and regulations.

Fisher’s exact test was used to evaluate the significance of differences in the severity of dislocation, patient age and incidence of AVN between the two groups. All statistical analyses were performed using the SigmaStat (version 3.1) program (Systat Software). A *p* value < 0.05 was considered significant.

## Results

There were 71 hips (52 patients). Age at operation ranged from 3 to 36 months, with an average age of 16 months; 42 patients were female and 10 were male. The left hip was dislocated in 40 cases and the right in 31; 25 patients had bilateral dislocation, but closed reduction of one dislocated hip was successful in six of those patients (Table 1).

**Table 1:** Demographic characteristics and clinical features of the patients and hips

**Total number of hips (patients)**	71 (52)
Average age at surgery (range of age at surgery)	16 months (3–36 months)
**Sex**	
Female	42 patients
Male	10 patients
**Side**	
Left	40 hips
Right	31 hips
Unilateral	33 patients
Bilateral requiring surgery on both sides	19 patients
Bilateral requiring surgery on one side only	6 patients

Before surgery, according to the Gage and Winter method, 57 hips were classified as being at the minus-one station while 14 hips were at the zero station. Among the patients who had hips at the minus-one station, 16 patients were aged less than 1 year, 15 were aged between 1 and 2 years and 11 were aged more than 2 years. Among the patients who had hips at the zero station, 6 patients were aged less than 1 year and 4 were aged between 1 and 2 years; none were aged more than 2 years. Preliminary traction was effective in 48 hips (68%, group 1), while it was not effective in the remaining 23 (32%, group 2). The results for groups 1 and 2 and the statistical differences between them are reported in Table 2.

**Table 2:** Results for group 1 (in which traction was effective) and group 2 (in which traction was not effective)

	Group 1	Group 2	*p* value
	Effective traction	Ineffective traction	
Total number of hips (number of patients)	48 (37)	23 (15)	
**Height of dislocation**			
Minus-one station	34	23	0.003
Zero station	14	0	
**Age distribution of patients at surgery**			
Patients < 1 years old (no. of hips)	22 (26)	0 (0)	< 0.001
Patents 1–2 years old (no. of hips)	15 (22)	4 (5)	
Patients > 2 years old (no. of hips)	0 (0)	11 (18)	
**AVN**	5 hips (10%)	8 hips (35%)	0.02

*p* value < 0.05 was considered significant

The height of dislocation was evaluated according to the Gage and Winter method

Among the 48 hips in which traction was effective (group 1), we observed five cases of AVN (three type II and two type IV). Among the 23 hips in which preliminary traction was ineffective (group 2), we observed eight cases of AVN (one type II, one type III, five type IV and one type V). The effectiveness of preliminary traction was statistically related to the lower incidence of AVN in group 1 compared to group 2 (*p* = 0.02).

We did not observe any difference in terms of final results between patients treated by traction in hospital and patients treated by traction at home.

## Discussion

The effectiveness of preliminary traction in reducing the incidence of AVN after closed or open reduction in CDH is still controversial. In 1972, Gage and Winter [25] reported that adequate traction reduced the incidence of AVN in a series of 154 cases of CDH treated with and without preliminary traction. In the same article, they proposed a classification method to assess the level of dislocation of the femoral head in order to evaluate the effectiveness of the traction. In 1979, Weinstein and Ponseti [14] reported that satisfactory results were obtained without using preliminary traction, including a low rate of AVN. Some years later, other authors [15, 28] agreed that prereduction traction was not useful; they reported that good results were achieved without preliminary traction and recommended abandoning this procedure. According to those authors, patients with CDH were safety treated with either closed or open reduction without preliminary traction. At the same time, Fish et al. [16] published a survey of 335 POSNA members regarding this issue. Most responders believed that traction reduced the incidence of AVN and enabled easier reduction; only 5% of those surveyed did not use preliminary traction; 31% were in favour of applying the traction at home, prolonging the traction time but reducing hospital costs.

During the subsequent decade, the authors of two different studies noted the utility of preliminary traction, including in older children [29, 30]. They used overhead traction in which the hip was flexed at about 100° and abducted at 20°. Those authors reaffirmed that overhead traction successfully achieved closed reduction in a high percentage of dislocated hips with a low incidence of AVN, including untreated congenitally dislocated hips in children 18–71 months old. They encouraged other orthopaedic surgeons to seriously consider the clinical utility of preliminary traction when treating CDH. On the contrary, at the same time, other authors did not find any benefits of preliminary traction in respect to increasing the possibility of closed reduction and reducing the incidence of AVN [17, 18]. They stated that “it cannot be proven that traction alters the outcome of developmental dislocation of the hip treatment, and hence there is only an anecdotal basis of its use” [18].

More recently, some authors have continued to recommend preliminary traction [11, 20–23, 31, 32]. Langenskiold et al. [20] compared two large series of patients with DDH treated with and without preliminary traction. They concluded that prereduction traction decreases the incidence of AVN of the femoral head in children aged 6–36 months and that traction represented the only difference between the two groups. In subsequent studies, other authors have reiterated the important role of prolonged preliminary traction with different methods to minimize the risk of AVN [11, 21, 23]. Park et al. [31], in a recent systematic review, concluded that “although there is a trend toward a decreasing use of traction, prereduction traction remains as a treatment tradition or a gesture toward medico-legal environment”. In the same year, Wicart et al. [32] reported a literature review evaluating the published results and complications of closed reduction in late-detected DDH. The authors concluded that the Petit-Morel method was the treatment of choice for children aged between 6 months and 3 years who were affected by idiopathic DDH. On the contrary, other authors in three different comparative studies that included a large series of dislocated hips reported that preliminary traction (overhead or longitudinal) did not decrease the failure of reduction or the incidence of AVN [12, 13, 19]. Schur et al. [33] even reported an increased risk of AVN when prereduction traction was used in a series of 70 children (82 hips).

The results reported by all the articles cited above are summarized in Table 3.

**Table 3:** Summary of literature data on the use of preliminary traction in the treatment of congenital dislocation of the hip

Authors	Journal, year of publication	Number of hips (traction vs no traction)	Use of preliminary traction	Incidence of AVN (%)	Length of follow-up	Recommendations for traction use
Weinstein, Ponseti (Iowa, USA) [14]	JBJS Am, 1979	10 vs 12 hips	Comparative	10% vs 10%	3.5 y	Not recommended
Kahle et al. (Utah, USA) [28]	JBJS Am, 1990	47 hips	No	3.5%	> 2 y	Not recommended
Fish et al. (Michigan, USA) [16]	JPO, 1991	Survey (POSNA)	Comparative	Not reported	Not reported	Recommended
Tavares et al. (Iowa, USA) [29]	JPO, 1994	27 hips	Yes	7.4%	Not reported	Recommended
Quinn et al. (Connecticut, USA) [17]	JPO, 1994	75 hips	Yes	6.7%	5.4 y	Not recommended
Daoud et al. (Algeria) [30]	JBJS Am, 1996	50 hips	Yes	10%	4 y	Recommended
Weinstein (Iowa, USA) [18]	CORR, 1997	Update	No	Not reported	Not reported	Not recommended
Langenskiold et al. (Finland) [20]	JPO, 2000	176 vs 86 hips	Comparative	2.8% vs 46.5%	13.1 y	Recommended
Kutlu et al. (Turkey) [19]	JPO, 2000	89 vs 65 hips	Comparative	4.5% vs 0%	5 y vs 2.5 y	Not recommended
Yamada et al. (Japan) [21]	JBJS Br, 2003	62 hips	Yes	1.6%	> 2 y	Recommended
Sibinski et al. (Poland) [22]	Int Orthop, 2006	107 vs 48 hips	Comparative	1.9% vs 14.6%	19.5 y	Recommended
Rampal et al. (France) [23]	JBJS Br, 2008	47 hips	Yes	2.1%	14.3 y	Recommended
Terjesen et al. (Norway) [11]	JBJS Am, 2014	90 hips	Yes	10%	51.6 y	Recommended
Schur et al. (California, USA) [33]	J Child Orthop, 2016	70 hips	Yes	35%	5 y	Not recommended
Sucato et al. (Texas, USA) [12]	JPO, 2017	276 vs 66 hips	Comparative	18% vs 8%	10.4 y	Not recommended
Park et al. (Korea) [31]	Ther Clin Risk Manag, 2018	Systematic review (683 hips)	Comparative	5–47% vs 0–72%	Not reported	Not recommended
Wicart et al. (France) [32]	J Child Orthop, 2018	Review	Yes	2% to 72%	Not reported	Recommended
Li et al. (China) [13]	JPO B, 2019	Systematic review (440 hips)	Comparative	14% vs 14.5%	3.1 y	Not recommended

To the best of our knowledge, the real effectiveness of preliminary traction, defined as the ability to pull the femoral head below the level of the triradiate cartilage, has never been radiographically assessed. In our study, we have verified, by radiographic monitoring, the real effectiveness of the traction in a series of 71 hips. In our patients, traction was effective in 68% of cases, while it was not effective in the remaining 32%. In the latter cases, the dislocations were more severe (femoral head at the minus-one station according to the Gage and Winter method) and the mean age of the children was higher; moreover, in this group of patients (group 2), we observed a statistically significantly greater incidence of AVN than in the group of patients for whom traction was effective (group 1). Therefore, we believe that the real effectiveness of traction itself should be verified before stating a definitive opinion on the real benefit in terms of decreasing AVN.

In the cases in which traction was not effective, we speculate that soft tissues were stiffer and muscles were more contracted than in the cases in which traction was effective. In the cases in which traction was not effective, femoral shortening osteotomy could be considered as a means to further reduce the AVN rate [34]. The reason for this may be that the dislocation was radiographically more severe in the majority of our cases in which traction was not effective, and the mean age of those patients was higher, regardless of whether the patient was treated in the hospital or at home. It is well known that muscle contraction is directly proportional to the extent of muscle lengthening and increases over time.

We agree with other authors [18] about the difficulty of radiographically assessing the effectiveness of overhead traction with the hip flexed at 90°. In our cases, we applied longitudinal traction with the hip flexed at 20°, so radiographic images were easily taken.

In our opinion, the length of traction time is another important factor to consider. In accordance with other authors, we believe that traction must be continued for at least 4 weeks to be effective [11, 21].

Finally, to reduce hospital costs, traction should be applied at home, as done in about half of our patients. We believe that, when compared to overhead traction, longitudinal traction is more easily managed at home, so we recommend a traction method that keeps the hip slightly flexed [26].

## Conclusions

According to the results for our study population, preliminary traction seems to reduce the incidence of AVN in patients surgically treated for congenital dislocation of the hip. However, our conclusions will need to be confirmed by further studies involving a control group of patients treated without preliminary traction. To evaluate the real effectiveness of traction, we support the use of longitudinal traction, which allows easy radiographic monitoring that can be performed at home, reducing hospital costs. The effectiveness of the traction is influenced by the severity of the dislocation and the age of the patient; it works better with less severe dislocations and in younger children.

## Data Availability

The data used and analysed during the current study are available from the corresponding author on reasonable request.
